# Twenty-second Annual Session of the Texas State Medical Association—Synopsis of Proceedings

**Published:** 1890-05

**Authors:** 


DANIEL'S

### TEXAS MEDICAL JOURNAL.

A Representative Organ of the Medical Profession, and an Exponent of Rational Medicine; devoted to the Organization, Advancement and Elevation of the Profession in Texas.

PUBLISHED MONTHLY.—SUBSCRIPTION $2.00 A YEAR.

Vol. V. AUSTIN, MAY, 1890. No. 11.

### TEXAS STATE MEDICAL ASSOCIATION—TWENTY-SECOND ANNUAL CONVENTION.

### Synopsis of Proceedings.

### FIRST DAY—MORNING SESSION.

OPERA HOUSE: FORT WORTH, TUESDAY, APRIL 22, 1890.

The meeting was called to order by the Chairman of the Committee of Arrangements, Dr. W. P. Burts; Rev. J. Morgan Wells, of the First Baptist church, invoked the Divine blessing on the labors of the Convention in an eloquent prayer, and was followe by the President, Dr. R. M. Swearingen, who, being introduced, declared the Twenty-second Annual Convention of the Association now open. Chairman Burts introduced Hon. W. S. Pendleton, Mayor of Fort Worth, who tendered the Association the freedom of the city, and a warm welcome, in one of his characteristic speeches. On behalf of the local medical profession Dr. Burts delivered a brief address, extending welcome to the delegates as guests of the local profession and of the city. President Swearingen responded to these words of welcome in a brief speech, after which he delivered the usual address, containing suggestions and recommendations for the "good of the order."

This was referred to a special committee of five, and Vice-President Sims—in the chair temporarily—appointed Drs. J. D. Osborn, T. D. Wooten, J. J. Burroughs, B. Sanders and J. M. Willis.

On call of Judicial Council the following members answered to their names and took their places, to wit: Drs. R. H. Harrison, Sr.; P. C. Coleman, M. D. Knox, F. S. White, E. M. Rabb, C. M. Ramsdell. The chair appointed to the vacancies, temporarily, O. Eastland, B. E. Hadra, J. W. Carhart, T. J. Field, W. P. Powell and E. J. Beall. Beall being absent and Carhart asking to be excused, the Council organized with ten members, and elected Dr. Harrison Chairman and Dr. Eastland Secretary. Dr. T. J. Bell, a member, reported later, and took his seat in the Council.

Dr. F. R. Martin offered the following resolution:

"WHEREAS, There seems to be a difference of opinion among the members present as to the tenure of office of the present Secretary, and an election is talked of for a successor; and

"WHEREAS, It is claimed, on the other hand, that there is no vacancy, the present incumbent having served only three years, since his election, therefore,

"*Resolved*, That the question be at once referred to the Judicial Council for decision."

The resolution was adopted.

The Secretary read his report as follows:

### REPORT OF SECRETARY.

FORT WORTH, TEXAS, April 22, 1890.

*R. M. Swearingen, M. D., President, and Fellows of the Texas S. M. A.:*

Your Secretary respectfully begs to report that in obedience to discretionary power given him by resolution at the last meeting, he had printed three thousand copies of the report on medical legislation, submitted by the chairman of the committee, Dr.

George Cupples, at that meeting, and that a copy was sent by mail as directed in the resolution, to the physicians of Texas.

At last report, there were carried on the roll of active members the names of 448 physicians; there were admitted to membership at the last meeting fifty-eight members, making a total membership of 506, as per the register. Since last meeting there have been six deaths, leaving the number 500. Of that number however several are no longer actual members, they having removed from the State and ceased paying dues. The deaths have been as follows: Drs. Ed. Randal, Galveston; J. B. Robertson, Goliad; J. M. Lewis, Mexia; R. B. White, Ennis; and Drs. A. E. and E. J. Carothers, of San Antonio. Doubtless our committee on necrology has preserved proper record of the respective dates, etc.

The removals from the State are Drs. Fred. Terrell, formerly of San Antonio; W. J. Pettus, late of Galveston; F. E. Yoakum, of Greenville. The address of several members has been lost through removal, and failure to report to the Secretary, to-wit: Dr. H. L. Taylor, late of Waco; F. M. Blakemore and W. W. Myers, late of San Marcos. If any member present knows their where-abouts, he will please report to the Secretary.

The library and archives of the Association are now cared for in the hall of the Austin District Medical Association at Austin.

There are now carried on the roll twenty-four auxilliary Associations, and there is no abatement in the interest in medical organization.

The correspondence of the Secretary's office has been unusual ly large, and courtesies have been exchanged with all the sister Associations. At last meeting there were admitted to honorary membership the following distinguished physicians: Drs. J. Harvey Reed, T. More Madden, of Dublin; J. L. Cabel, of Virginia, recently deceased, and Dr. T. F. Noyes, of Detroit.

The Seventh Decennial Convention for the revision and publication of the Pharmacopœia of the United States of America, will be held in the city of Washington, D. C., on the first Wednesday in May, prox., the seventh day, at noon. This Association is entitled to representation, and his been called on by the President, Dr. Robert Armory, to appoint delegates, not over three. The necessary credentials have been forwarded, to-wit: Certificate of incorporation, signed by the Secretary of State. The Secretary has been provided with two blank certificates to be filled out and given the delegates selected at this meeting.

In accordance with instructions as per resolution adopted at the last meeting, authorizing the President and Secretary to secure a charter from the State, I have the honor to report that the duty was promptly performed. A copy is on file in the office of the Secretary of State, and the articles of incorporation, together with the names of the trustees appointed by the President for the first year, were published in the Transactions. It will be necessary to elect a new Board of Trustees at this meeting, as the term of office is only one year.

Respectfully submitted,

F. E. DANIEL, M. D., Secretary.

SECRETARY'S FINANCIAL REPORT.

*F. E. Daniel, M. D., Secretary, in Account with Texas State Medical Association:*

DEBTOR.

| | | |
|---|---|---|
| 1889–90. | To balance as per last report. . . . . . . . . | $ 18 61 |
| | To amount, dues paid by members, to-wit: Drs. O. J. Kendall, W. M. Powell, T. J. Bennett, J. F. Dean, J. S. Lankford, W. A. Morton, J. C. Jones, T. S. Burke, J. M. Burleson, F. H. Tucker, and Jas. Cowling. Eleven at $5 each . . . . . . . . . . . . | 55 00 |
| | Dr. J. Cummings . . . . . . . . . . . . . | 10 00 |
| | Cash from the sale of Transactions . . . . . | 5 05 |
| April 20. | Dr. J. A. Davis, with application . . . . . . | 10 00 |
| | Total . . . . . . . . . . . . . . . . . . | $98 66 |

CREDIT.

| | | |
|---|---|---|
| | By cash, stationery last meeting (no voucher) | $ 85 |
| | By cash paid advertisement for bids, (voucher 1) | 35 |
| | Cash paid, acknowledgement three signatures to charter, (voucher No. 2) . . . . . . . | 1 50 |
| | By cash paid stamps on 75 copies Transactions, (voucher No. 3) . . . . . . . . . . | 6 75 |
| | By cash paid, expressage, (voucher 4) . . . | 45 |
| | Cash paid for stamps for President's letter (voucher 5) . . . . . . . . . . . . . . | 5 00 |
| | Cash paid for stamps on Transactions returned (no voucher) . . . . . . . . . . . | 63 |
| | Cash paid for stamps on single Transactions as ordered, (no voucher) . . . . . . . . . | 1 10 |
| | Cash paid for stamps for Cupples' report on Medical Legislation, 3000 copies (vouchers 6 and 9) . . . . . . . . . . . . . . . | 30 00 |
| | Cash paid for stamps and postage used in office during the year (no vouchers) . . . | 5 00 |
| | Clerical work on above, 3 days (voucher 7) . | 7 50 |
| | Cash paid for printing 600 President's letter, 3000 Cupples' reports, 3000 envelopes, voucher 8 . . . . . . . . . . . . . . . . . . | 42 50 |
| | Cash paid for drayage, no voucher. . . . . | 50 |
| April 19, | Voucher 10, certificate of incorporation Secretary State . . . . . . . . . . . . . | 1 00 |
| | Total.. . . . . . . . . . . . . . . . . . . | $103 13 |
| | | 98 66 |
| | Balance due Secretary . . . . . . . . . . . | $ 4 47 |

E. & O. E., April 22, 1890.

F. E. Daniel, M. D.- Secretary.

The report was on motion received, and referred to a special committee consisting of Dr.s F. R. Martin, H. C. Ghent and J.

T. Wilson. The committee after careful examination reported it correct in every detail. (See report.)

The Treasurer then submitted his report as follows:

TREASURER'S REPORT.

*Dr. J. Larendon, Treasurer, in Account with Texas State Medical Association:*

RECEIPTS.

| | | |
|---|---|---|
| 1890 | | |
| Apr. 16. | To cash balance on hand as per last annual report . . . . . . . . . . . . . . . . . . | $ 44 91 |
| " " | To cash collected from members, dues up to this date . . . . . . . . . . . . . . . . | 1,404 50 |
| | Total . . . . . . . . . . . . . . . . . | $1,449 41 |

DISBURSEMENTS.

| | | |
|---|---|---|
| 1889. | | |
| Apr. 23. | Cash paid J. R. Briggs, (prize essay) . . . | $ 100 00 |
| " " | " " J. F. Y. Paine, (due bills) . . . . | 60 00 |
| " " | " " F. F. Daniel, (Sec'y salary) . . | 200 00 |
| " " | " " Publishing committee . . . . . . | 300 00 |
| " " | " " J. Larendon, (Treas. salary) . . . | 100 00 |
| " " | " " J. Larendon, (for postage) . . . . | 10 00 |
| " " | " " George Cupples, for postage on report in surgery . . . . . . . . . . . . | 14 00 |
| May 10. | Cash paid A. C. Gray for printing books, receipts, paper and envelopes . . . . . . . | 9 00 |
| July 24. | Cash paid fee for charter . . . . . . . . . | 10 00 |
| Sept, 30. | Cash paid E. Von Boeckmann for 650 copies of Transactions and postage . . . . | 567 00 |
| " " | Exchange . . . . . . . . . . . . . . . . | 1 41 |
| 1890. | | |
| Apr. 16. | To cash balance on hand . . . . . . . . . . | 78 00 |
| | Total . . . . . . . . . . . . . . . . . | $1,446 41 |

Respectfully submitted,

J. LAREDON, Treasurer.

Houston, April 16, 1890.

The report of the Treasurer was referred to Drs. H. A. West, L. J. Graham and W. P. Perkins, who, after examination, reported it correct.

The Publishing Committee then submitted their report, as follows. It was referred to Drs. F. A. Schmitt, F. D. Thompson and J. C. Loggins:

REPORT OF THE PUBLISHING COMMITTEE.

*To the President and Members Texas State Medical Association:*

Your Committee on Publication beg leave to respectfully report that in obedience to instructions and in accord with custom, they advertised for bids for printing the Transactions. There were two only, and that of Eugene von Boeckmann being the lowest and best, the contract was awarded him. His bid was ninety-three cents per page (against $1 last year), and twenty-seven and a half cents per copy for the binding for cloth binding, the same as that for which was paid formerly 35 cents; no charge for pamphlet covers. The committee had 500 copies bound in the cloth, and 150 in paper, 650 copies in all. The total cost of the edition was $507, or 78 cents per copy. The work was promptly and satisfactorily done, and the Transactions were in the hands of readers earlier than usual, and ahead of nearly all the sister Associations. The bill, including $60 postage paid by the publishing house, was paid, after approval, by sight draft on the Treasurer, and does not, therefore, figure in the Secretary's financial report. A copy was, of course, sent to each member, and to the honorary members, home and foreign; seventy-five copies were afterwards sent to the medical press, boards of health, colleges, libraries, etc., the postage on which, as on single volumes, as it became necessary to issue them, was paid by the Secretary.

Your committee submit herewith the Transactions, and trust their service has been satisfactory. In their opinion the work is as well done, and done as cheaply, as it could have been done in Chicago or St. Louis; and it is the cheapest and one of the best ever gotten out for the Association. Respectfully submitted,

F. E. DANIEL, Chairman,
T. J. BENNETT,
J. W. McLAUGHLIN,
Committee.

The committee reported as follows:

"Your committee to examine the report of the Publishing Committee have carefully examined it according to instructions, find it correct in every particular, and recommend its adoption."

F. A. SCHMITT, Chairmam,
LOGGINS,
THOMSON.

Dr. Ghent offered the following resolution, which, on motion, was adopted:

*Resolved,* That the Secretary be instructed to procure the names, as far as possible, of all the deceased members of the Texas State Medical Association, from its organization to the present time, who were in good standing at the time of death, with the postoffice and county of each, and the year admitted as members and the year of death, and have the same published at the close of our next yearly Transactions under the caption, "In Memoriam."

On call of committees President Swearingen stated the reasons why he had not appointed the Committee on State Board of Health provided for by resolution at last meeting. The Committee on Necrology, on Medical Legislation and on Report of Surgical cases were called in order, and as the chairman of each was absent, no report was made.

Dr. T. C. Osborn, an honorary member, offered the following resolution:

"WHEREAS, The Constitution of the Texas State Medical Association limits the number of its Vice-Presidents to only three of its trusted members; and

"WHEREAS, In the 2d section of article 5 the only prescribed function of the Vice-President is that of serving as President in case of the absence of the President from the meeting; and

"WHEREAS, Important interests of the Association would be better subserved and more enhanced, if more energetic laborers were in the field of its work, and especially, if the honored Vice-Presidents were endowed with other and additional function than that of mere figure head; and

"WHEREAS, The magnitude of work would be greatly simplified and systematically methodized, and would redown to the vitality and push of professional interest, if the State were divided in four quarters, with one Vice-President for each quarter, who shall be charged with the duty of urging the organization of county medical societies where none exist, and as a center of reference for societies already established, and to whom monthly or quarterly reports of progress should be made, and written reports of the Vice-President made annually to the Association, embodying such or so much of the county reports as may seem to either of the Vice-Presidents worthy of utility and commendation; therefore, be it

*Resolved*, That the Constitution of the Texas State Medical Association be so amended in section 1, article 4, as to read *four* instead of *three* Vice-Presidents, and that section 2 of article 4 be amended to read: "It shall be the duty of the Vice-Presidents to preside in case of the absence of the President; in the rank in which they are numerically recognized; and that one Vice President shall be elected from each quarter of the State, such quarters to be designated in the By-laws of the Association; and that each Vice-President is charged with the duty, either in person or by correspondence, of urging the organization of medical societies in each of the counties over which he is the central figure, and to assist and encourage those societies already established, receiving monthly or quarterly reports from all of them, and embodying in his written annual report so much of the county reports as he may deem of professional interest and utility to the Association.

"*Resolved, further*, That, for the purpose of convenience and general advancement of State professional progress, the Texas State Medical Association adopt as one of its By-laws a division of the State into four quarters, the first line beginning east on the 31st parallel of latitude, running west across the State; and the second line beginning north on the 98° of longitude, running south across the State, the lines so meandering as to include entire counties in both directions; the quarter thus designated and established to be numbered first, second, third and fourth; the first being in the southeast, the second in the northeast, the third in southwest, and the fourth in northwest section of the State.

On motion, Dr. Osborn's resolution was received, and a motion was made to refer to a special committee for immediate action and report. Dr. A. M. Douglas offered as a substitute that the report be referred to the Publishing Committee, with instructions to incorporate it in the volume of Transactions, and that it be acted on at the next meeting. The point was raised that all proposals to change the Constitution must lie over one year.

Dr. Osborn, the mover of the resolution, pointed out that there is no such requirement in the Constitution and By-Laws of the Association, and the President decided accordingly.

Dr. Ward said that there is an unwritten law which requires it, and that all parliamentary bodies are governed by it. Custom has become the law.

Dr. Stout moved to lay the resolution on the table. No second to his motion.

The question being called, the vote was on Dr. Douglas' substitute, which was adopted, and the resolution was so referred.

Dr. G. H. Lee, by permission, read an address by Dr. Joseph Jones, of New Orleans, to the survivors of the medical corps of the C. S. Army. No action was had upon it.

Adjourned to 2 p. m.

---

### FIRST DAY—AFTERNOON SESSION.

TUESDAY, APRIL 22, 1890.

When the meeting was called to order, the Section on Practice was called. The President resigned the chair to Chairman D. M. Ray; Dr. O. Eastland, Secretary of the Section.

Dr. F. A. Schmitt, of Shulenburg, read a paper giving an account of how he was inoculated in some mysterious manner in delivering a healthy woman in a premature labor. He had an abrasion on his finger, and through this his system was invaded by a violent blood poison, which came near taking his life. It was a most interesting paper. Paper received and referred to Publishing Committee.

Dr. J. D. Burch read a paper giving an account of the epidemic of cerebro-spinal meningitis, which prevailed in and about Aurora last summer. The paper was received and referred as usual, but with a vote of thanks to the author and instructions to the Publishing Committee to publish it in the Transactions.*

The reading of this paper awakened a lively discussion, which was participated in by many members, some claiming the disease to be contagious, and some the reverse.

Prof. Cain, of Nashville, was requested to give his views of the subject, but politely asked to be excused. All the speakers complimented Dr. Burch's paper.

Dr. J. W. McLaughlin read an excellent paper, entitled "An Explanation of the Phenomena of Contagion and Immunity from Disease based on the Action of Physical and Biological Laws."

This paper was listened to with deep interest, and a lengthy and spirited discussion followed.

Dr. Sears moved that it be received and referred to the Publishing Committee, and that a vote of thanks be tendered the author. Adopted.

Adjourned to 9 a. m., Wednesday.

F. E. DANIEL, Secretary.

Approved:

R. M. SWEARINGEN, President.

### SECOND DAY—MORNING SESSION.

WEDNESDAY, April 23, 1890.

Prayer by Rev. R. H. Nalle, of the Presbyterian church. Minutes of previous day's proceedings read and approved.

Dr. Daniel, the Secretary, rose to a point of personal privilege, and asked to be permitted to speak on a subject of importance to the welfare of the Association. He said that the question as to the tenure of office of the present Secretary was in dispute, and

*The publication of any paper read is discretionary with this committee, unless publication is thus requested.—ED.

had been referred to the Judicial Council, at the beginning of the first session. They had deliberated on it twenty-four hours without reaching a decision, and now in the interest of harmony, he wished to settle the question. He had not sought the office; it had been tendered him on the death of Dr. Burt, and he had been urged by the then President to serve pro tem., it being represented to him that it was the wish of a large number of leading and representative men who had asked that the appointment be made. He declined to accept the appointment unless the President, Dr. Nott, would appoint him for all of Dr. Burt's unexpired time, which would have been up to the present meeting. Dr. Nott decided that he could make the appointment only up to the next meeting (at Austin in 1887) and stated in his letter that the office would then be permanently filled by an election. The request to serve temporarily was again urged, and accepted. At Austin an election was held, and he was elected. He had asked no man to vote for him, and had served because it was the wish of the Association. As, at the time of election nothing was said of any unexpired time, he presumed, and his friends now hold, that his election was for five years, inasmuch as the Constitution says the Secretary shall be elected for the term of five years; not that an election for Secretary shall be held every five years. He had served, to date, three years since his election. He had served them to the best of his ability, and as there had never been any complaint, he flattered himself that his services had been satisfactory. Now, there seems to be a division of sentiment as to whether or not his term has expired, and some good men conscientiously think that by precedent in the succession of Burt to Bibb on the latter's resignation at the end of two years, an election for Secretary should be held now and here. He had contended for it—not because he wanted the office, but on principle, and to vindicate the law, which, to him, was unmistakable, that he was elected for five years. He had abored many years in the interest of organization, and towards the younger societies he felt as a painter feels towards the creations of his own hand and brain. He was particularly devoted to the State Association, and had expended his best efforts in building it up; had striven for the advancement, elevation and purification of the medical profession in Texas; and now, in view of the foregoing, and as an earnest that he values harmony in the ranks, and the prosperity of the Association far above the pittance paid for his services, he tendered his resignation, and requested that a successor be elected at this meeting.

The resignation of Dr. Daniel, as Secretary, was accepted, and the President re-appointed him, and requested him to serve till a successor could be elected; which would be on the following day.

The Judicial Council came in to report on this subject, but Dr. Daniel stated that he had, by his resignation, anticipated any action on their part; that the matter had been withdrawn, and asked that they make no report. Dr. Becton arose and said that Dr. Daniel's action was manly; was magnanimous, and greatly to be commended. He agreed with Dr. Daniel that the matter was now out of the hands of the Council, and that as Dr. Daniel had resigned, in the interest of harmony, he thought it very unwise to permit the Council to make any report whatever. In either event—whether they sustain him in the claim of two more years, [which was now withdrawn] or the reverse, a report would be a fire-brand which would effectually defeat the object of his magnanimous withdrawal.

The President held that now that all personalities had been elimated from the subject, the Council should report and forever put at rest the question, whether in similar cases the Secretary has to serve five years or only the unexpired term of his predecessor.

At the further request of the ex-Secretary and Dr. Becton, the President conceded the point, and the matter was dropped.

Dr. J. D. Osborn, Chairman of the committee on the President's recommendations, presented the following report:

REPORT OF COMMITTEE ON PRESIDENT'S SUGGESTIONS AND RECOMMENDATIONS—J. D. OSBORN, CHAIRMAN.

"Your committee on the President's message, would respectfully recommend as to his suggestions to Article V. of the By-

Laws: 1st. Total abolition of all per capita tax upon members of County Societies, and that the representation from each County Society be the same as now, and said delegate elected by said County Society, must present his credentials and become a member of the State Association, if not already one, by payment of his initiation fee, ($5.00) as prescribed by the Constitution, and we would further suggest that the Constitution and By-Laws be so amended as to include District Medical Society's representation on same basis. Upon complying with these recommendations the delegate becomes a member of the Association, and is entitled to all its benefits and privileges; and further, the delegate representing a society shall furnish to the Secretary of this Association a roster of his society, which shall appear in the Transactions, under the head of, 'District and County Societies.'

"We cordially endorse all of that part of the President's message in reference to a State Board of Health, and recommend that it be incumbent on the present President to appoint said committee; and we would recommend that the committee be also authorized to secure the passage of a satisfactory law regulating the practice of medicine, and in case of failure to do the same, they can at least secure the repeal of the present law, creating District Examining Boards, and to secure the passage of a law allowing none to practice medicine except those who are graduates of regular chartered schools. This is in nowise intended to be retrospective, nor to disturb the legal qualification of those already qualified, according to existing laws. We would further suggest that in order that our 'full measure of usefulness' be placed upon the firmest foundation, and that our financial standing be assured, that the Treasurer of the Association be the sole guardian of the badges, and issue them to members only who have paid their dues; this being in full accord with the practice of the American Medical Association; and that a copy of the Transaction be sent to them, only, who have paid their dues; all of which is respectfully submitted for the final consideration of the Association."

THOS. D. WOOTEN, M. D.,
J. J. BURROUGHS, M. D.,
BACON SAUNDERS, M. D.,
J. D. OSBORN, M. D.,
Committee.

Dr. Ward moved that the report be received and spread upon the minutes as a part of the unfinished business to be taken up at the next meeting.

Dr. Gilbert moved to refer the report back to the committee with the request to divide it, so that that part which can have immediate attention can be acted upon, as there was a part which contemplates a change in the Constitution, and which, he supposed must lie over one year.

Dr. Osborn explained that the idea of his committee was that the committee proposed in the report, should be appointed and go to work at once.

Dr. T. H. Nott offered the following as a substitute to Dr. Gilbert's motion: "I move that all the report of the committee upon the President's message be adopted except that part related to the contemplated change in the organic law, which shall be received and laid over for the action of the Association at its next regular meeting."

On motion Dr. Nott's substitute was adopted.

Committee on State Board of Health—appointed in accordance with suggestions of Committee on President's Recommendations —Dr. A. M. Douglass, Chairman, Osceola, with discretionary powers to appoint four associates.

The Judicial Council reported favorably upon the applications of forty-eight physicians for membership, as per list published elsewhere, and submitted also the following resolution, which on motion was adopted:

"WHEREAS, The Judicial Council find great difficulty in the thorough investigation of the fitness of applicants for membership in this Association,

"*Resolved*, That in future every applicant must without fail accompany his application with his diploma or other acceptable documentary evidence of graduation."

R. H. HARRISON, Chairman.

O. EASTLAND, Secretary.

At this point the Section on Obstetrics was called. The Chairman, Dr. S. D. Thruston, being absent, Dr. J. T. Wilson was requested to act as Chairman. He appointed Dr. B. Saunders Secretary.

Dr. E. J. Ward read a paper entitled, "Is Dentition a Factor in Infantile Disease?" Paper received and referred to Publishing Committee.

Dr. Q. C. Smith came forward and exhibited some obstetric instruments which he had devised or modified, and explained his method of operating, especially in case of placenta prævia.

Dr. West said it reminded him of the man who had an improved speculum which could be used at will, either as a speculum or as a tongue—depressor; and asked Dr. Smith to point out more specifically the advantages in his method in placenta prævia, which he did. This led to a discussion of placenta prævia.

Dr. Carhart read a paper on the necessity of special study on the part of the general practitioner. Received and referred as usual. A paper by Dr. W. T. Evans, on Ante-Partem hemorrhage, was read by title and referred.

Adjourned to 2 p. m.

---

SECOND DAY—AFTERNOON SESSION.

WEDNESDAY, April 23.

Section on Surgery and Anatomy called. Dr. W. L. York, Chairman, presiding. Dr. W. B. West, Secretary.

Dr. York read his address in surgery. Referred; as were also the following papers: By Dr. H. L. Fountain, "Five Cases Cranial Surgery." By Dr. B. F. Brittain, a paper on "Hemorrhoides." By Dr. W. M. Powell, report of a case of "Compound Comminuted Fracture of Leg with Gangrene."

Dr. W. B. West exhibited a child suffering from spondylitis, on which he was using a Sayres' jacket and jury-mast, and in which there had been great improvement. Dr. Gilchrist brought forward a patient with an ulcerating disease of the neck, which he supposed to be epithelioma, and asked for an opinion by the physicians present.

Dr. Nott related the history of a case occurring in his practice—no discussion.

Dr. B. F. Brittain submitted a paper giving an account of removal of a carcinomatous breast. Read by title and referred.

Dr. Q. C. Smith exhibited some rectal instruments of his own invention, and described his method in hemorrhoides and other affections of the rectum.

Dr. R. H. L. Bibb, of Mexico, related how he had removed a tracheotomy tube from the throat of a patient, in whose trachea it had, by negligence, been left a long time. Exhibited the tube and made some interesting remarks on the case, which, on motion, he was requested to put in writing and send to the Secretary for publication.

Section closed, and that on State Medicine and Public Hygiene was called. The Chairman, Dr. E. J. Ward, presiding.

Dr. L. J. Cunningham read a paper on "How to obtain a Board of Health System for Texas." Received and referred to Publishing Committee. No discussion.

Section on Gynecology called. Dr. H. K. Leake, Chairman, presiding; Dr. Brooks, Secretary. Dr. Leake read his report or address, which was received and referred as usual, with instructions to publish in the Transactions, and a vote of thanks was tendered the author. This paper elicited an interesting discussion and was much complimented.

Section on Practice recalled. Dr. Ray in the chair. Read by caption and referred a paper on "Malarial Hematuria," by Dr. G. M. Stevens; one by Dr. A. W. Achison, on "The Best Way to Use the Capsule." Dr. H. A. West read a paper, entitled "Clinical Notes on Medical Cases at Sealy and St. Mary Hospitals." Received and referred. Adjourned.

F. E. DANIEL, Secretary.

Approved:

R. M. SWEARINGEN, President.

[The President's address was delivered at the Opera House on Wednesday evening, 8:30.]

### THIRD DAY—MORNING SESSION.

THURDAY, April 24, 1890.

The meeting was called to order at 9:30 a. m., by the President. Prayer by Rev. R. C. Armstrong. A quorum present at roll call. Minutes of yesterday read and approved.

Dr. A. Sims offered a resolution providing for the appointment of a committee by the President to regulate the length of papers and the number of words in each, to be read in any section, and providing that any paper which would require over twenty minutes in reading, be read by caption only.

Dr. Smith moved to amend by reading such papers by abstract, instead of by title. The resolution was adopted as amended.

Dr. J. W. Carhart offered a resolution to the effect that the Association is remiss in its duty with regard to deceased members, and that the President be required to name a time on the first day of the session each year, for memorial services to be held for deceased members, said time not to exceed one hour, and such services to be the order of the day and take precedence over all business.

Dr. Gilbert offered as amendment that the report of the Committee on Necrology be the order of the hour instead of the order of the day. No second.

Dr. Osborn moved as amendment to Dr. Carhart's resolution that a page in the Transactions be devoted each year to a record of deceased members.

Dr. C. J. C. King moved to amend by making it "a page or more, as required."

The vote was taken and Dr. Carhart's resolution, as amended, was adopted.

Dr. H. K. Leake offered the following resolution:

"*Resolved*, That a Committee on Microscopy and Pathology be added to the standing committees of this Association, to the end that these branches of study be thereby fostered, and the wants of the profession in these directions met by competent investigators. Further, that such committee be known as the Committee on Microscopy and Pathology of the Texas State Medical

Association, and shall consist of three members, to be nominated by the Nominating Committee." Adopted.

Section on Medical Jurisprudence and Psychology was called. Dr. A. D. Burroughs, the Chairman, was absent, and the President asked Dr. D. R. Wallace, to preside. Dr. Wallace then read a paper on "Psychology, Past, Present and Future." At the conclusion of the paper and before any action could be had on it, Dr. Wallace stated that it was not intended for publication, and he did not wish it to go to the Publishing Committee. The President stated the law on the subject, which makes all papers presented the property of the Association, but, on motion, Dr. Wallace was permitted to withdraw the paper.

The Nominating Committee, composed of one representative from each county, was then called and retired for work.

[List omitted here.—Ed.]

They organized by electing Dr. S. H. Stout, Chairman, and Drs. J. E. Erwin and P. C. Coleman, Secretaries.

Dr. J. T. Wilson offered a resolution, authorizing Dr. McLaughlin, in view of the unusual originality and merit of the paper read by him the first day, and that he might secure priority in the promulgation of his views, to have it published prior to the publication of the Association volume of Transactions. The resolution was adopted.

The Sections, respectively, on Ophthalmology and Otology, Dermatology and Electro-Therapeutics were called, but the chairman of each was absent, and no papers were presented in either section. Adjourned to 2 p. m.

———

THIRD DAY—EVENING SESSION.

Thursday, April 24, 1890.

Meeting called to order at 2 p. m. Section on Practice recalled, as the time allotted the first and second days was not sufficient to get through the business of the Section. Dr. Ray, the Chairman, presiding, read his address in medicine, the subject being the "Germ Theory of Disease." Paper received and referred. Quite an animated discussion followed, in which Dr. Sears, of

Waco, took issue with the writer and denied the influence, in causing diseases, attributed to so-called "germs." Dr. Terhune and Prof. Cain (of Nashville) participated in the discussion, the latter sustaining the theory. Dr. H. A. West endorsed Dr. Cain and spoke at length on germ causation, and said that until now he did not know that there was an educated physician in Texas who refused to accept the theory.

Dr. Q. C. Smith read a paper on the "Treatment of Consumption." Paper received and referred.

A paper by Dr. T. More Madden, an honorary member, resident of Dublin, Ireland, was read by title and referred, and a vote of thanks to Dr. Madden was recorded. Also a paper by Dr. C. M. Ramsdell, and one by Dr. F. E. Yoakum, read by title and referred. The title of Dr. Yoakum's paper was "Alcoholics in Disease."

The Nominating Committee now reported as follows:

For President—Dr. W. P. Burts, of Fort Worth, by acclamation.

*Dr. J. M. Fort, of Paris, First Vice-President.

Dr. M. D. Knox, of Hillsboro, Second Vice-President.

Dr. W. W. Reeves, of Wills Point, Third Vice-President.

Dr. F. E. Daniel, of Austin, Secretary. [Re-elected for five years.]

Dr. J. Larendon, of Houston, Treasurer. [Re-elected for five years.]

Section on Practice of Medicine—Dr. H. A. West, Chairman, Galveston.

[Dr. West has appointed Dr. B. F. Church, of Austin, Secretary of the Section.]

Section on Obstetrics and Diseases of Children—Dr. J. W. Carhart, Chairman, Lampasas.

Section on Surgery and Anatomy—Dr. L. S. Gilbert, Chairman, Sulphur Springs.

Section on Medical Jurisprudence and Psychology—*Dr. J. T. Wilson, Chairman, Sherman.

---

*Resigned as soon as announced, and Dr. King, of Waco, was appointed First Vice-President. Dr. Jno. Preston was appointed Chairman of the Section on Med. Jurisprudence.

Section on State Medicine and Public Hygiene—Dr. R. Rutherford, Chairman, Houston.

Section on Gynecology—Dr. C. F. Paine, Chair'n, Comanche.

Section on Ophthalmology and Otology—Dr. Frank Gray, Chairman, Fort Worth.

Section on Dermatology—Dr. W. T. Baird, Chairman, Dallas.

*Publishing Committee—Dr. F. E. Daniel, Secretary, ex-officio Chairman; Dr. J. W. McLaughlin and Dr. T. J. Bennett.

Committee on Collection of Reports of Surgical Cases—Dr. Geo. Cupples, Chairman, San Antonio.

Committee on Medical Legislation—Dr. Geo. Cupples, Chairman, San Antonio.

[This committee is continued, except that Dr. R. H. Harrison was put in place of Dr. J. H. Pope, removed from the State.—Ed.]

Committee on Necrology—Drs. J. W. Carhart and T. D. Wooten.

[The Committee on Necrology, according to the By-Laws, should consist of a member from each county. The By-Laws also require the Nominating Committee to elect Secetaries of Sections—which is never done.—Ed.]

Delegates to the American Medical Association, May, 20—Drs. J. W. Miller, G. D. Bond, J. E. Gibson, R. Rutherford, R. H. Harrison, Sr., H. C. Ghent, E. P. Becton, F. E. Yoakum, C. F. Paine, E. J. Beall, T. D. Wooten, Geo. Cupples, R. A. Williamson, B. F. Eanes, F. E. Young, J. H. Sears, J. T. Wilson, B. Sanders, W. P. Burts, W. A. Duringer, J. J. Williamson, O. Eastland, J. D. Osborn.

Delegates to the Tenth International Medical Congress, at Berlin, in August, 1890—Drs. T. J. Tyner, O. Eastland, J. F. Y. Paine, Geo. Cupples, P. C. Coleman, A. W. Fly.

Delegates to Seventh Decennial Convention for Revision of the U. S. Pharmacopœia, at Washington, May 7, 1890—Drs. A. N. Denton and Q. C. Smith, Austin, with Drs. F. E. Daniel and T. J. Bennett, as Alternates.

---

*Committee continued, or reappointed.

Members of Judicial Council for three years—Drs. J. C. J. King, W. P. Powell, H. L. Fountain, *B. Sanders.

Trustees under the Charter—the original Board re-appointed as follows: Drs. J. F. Y. Paine, S. R. Burroughs, O. Eastland, W. L. York, E. L. Thompson, A. G. Clopton, G. W. Kerr, J. H. Sears, and A. N. Perkins.

The time and place for next meeting—Waco, fourth Tuesday in April, '91.

Committee of Arrangements—Dr. J. H. Sears, Chairman.

The report of the Nominating Committee was, on motion, received and adopted.

Dr. H. A. West moved that a committee consisting of Drs. George Dock, Chairman, and J. W. McLaughlin be appointed, to be known as the Committee on Bacteriology, Biology and Microscopy. The motion was adopted.

The newly elected officers were escorted to the stand and introduced, each making an appropriate speech.

President Swearingen, in yielding the gavel to his successor, President Burts, made an appropriate speech, returning thanks, etc., as did the latter on assuming the duties of his office.

President Burts then called for a report from the Committee on Surgical cases. The Chairman, Dr. Cupples, had not been able to get his report ready, in consequence of other and heavy labors. The same with regard to the Committee on Legislation; but promised to have a report of each at next meeting.

The delegates were invited to a banquet at the Pickwick Hotel on that evening, Thursday, April 24, and it was a most elegant affair. The ladies accompanying the delegates were present, and added much to the enjoyment of the occasion.

Adjurned to Friday morning.

F. E. DANIEL, Secretary.

Approved:

S. R. BURROUGHS, Acting President.

---

*Resigned when announced. No appointment made to date.—ED.

### FOURTH DAY—MORNING SESSION.

---

FRIDAY, April 25.

The Convention met at Opera House, at 9:30. President Burts being called away on business, asked ex-President S. R. Burroughs to take the chair, as none of the Vice-Presidents were present. The minutes of yesterday were read and approved.

The following resolution, offered by Dr. J. H. Sears, was adopted:

"WHEREAS, Our efficient Treasurer has served us for nineteen years, the first ten of which was without compensation; and,

"WHEREAS, There is no provision in the By-Laws for any remuneration for this officer, but at each meeting a small appropriation has generally been made,

"*Resolved*, That the sum of $150 be set aside for his salary each year out of the funds of the Association, and that $10 be allowed him for postage each pear, as heretofore."

The committee appointed to examine the Secretary's financial report, submitted the following:

"Your committee appointed to examine the Secretary's report, beg to state that they have performed that duty, and find the report correct. But in reference to one item, we would respectfully ask the Secretary to explain voucher No. 7, where he paid $7.50 for clerical work."

F. R. MARTIN,
T. J. WILSON,
H. C. GHENT.

Report received.

Ex-President Swearingen arose, and said that every year it became necessary for the Secretary to pay out money for such labor as wrapping, tying and stamping the several-hundred volumes of Transactions, and for folding, enveloping and stamping the large number of circulars sent out annually from his office. In this instance there were 3000 pamphlet circulars (Report of Committee on Medical Legislation) to be folded, put in envelopes, stamped and addressed. That the Secretary had gotten off 1000, when he was taken down with an illness that lasted until a few days before this meeting; and that in order to get off the remaining 2000, it had become necessary to employ assistance, as he was in bed, and that he had authorized it.

This explanation was satisfactory. Dr. Ghent then wanted to know by what authority the Publishing Committee were paid each year $300, stating that he was not aware of the existence of such authority, etc. Whereupon, at request of ex-Prisident Swearingen, who remarked that as President of the Association, he would not have been apt to issue a warrant on the treasury for money not authorized by law, the Secretary then read the ordinance by Dr. Sears, on page 303, Transactions 1889, which was adapted at Austin, in 1887. This seemed to be satisfactory, and business was proceeded with.

Dr. H. A. West offered the following resolution:

"WHEREAS, It has become notorious that some Chairmen of Sections duly elected, fail entirely in the performance of the duty incumbent on them, both in preparing reports, and in absenting themselves from the meetings of the Association; and,

"WHEREAS, An important By-Law of the Constitution, requiring the Secretaries of the various Sections to be notified as to the titles, or have the papers sent to the Secretary at least one month before the meeting which is to act upon them, and to give due notice of the same; and,

"WHEREAS, The general Secretary is unable fully to report the discussions occurring in the Section work, which ought to constitute the most interesting part of the proceedings,

"*Resolved*, That every Chairman-elect shall notify the general Secretary, at least one month after election, of his acceptance or otherwise of the position. Should any Chairman fail to furnish such notice, the President shall then appoint a new Chairman.

"*Resolved*, That this Association *insist* upon the literal performance of the requirements of the by-laws, referring to the transmission of papers to the Secretaries of Sections, and publication thereof.

"*Resolved*, That the general Secretary be authorized and empowered to select a competent stenographer and type-writer to assist in reporting discussions and proceedings of the Association at each meeting."

Dr. A. M. Douglas offered to amend by requiring the general Secretary to give notice to each Chairman of his election.

Dr. West spoke strongly in support of his resolution, and on motion it was adopted as amended by Dr Douglas.

After the usual vote of thanks to the citizens, the Committee of Arrangements, the ladies, etc., and to the Natatorium Company for aninvitation to use their baths, the Association adjourned to meet in Waco, the fourth Tuesday in April, 1891.

W. P. BURTS, President.

F. E. DANIEL, Secretary.

